# Supplementary material for: Symbiodiniaceae and Ruegeria sp. Co-Cultivation to Enhance Nutrient Exchanges in Coral Holobiont
Source: Microorganisms. 2024 Jun 17;12(6):1217. doi: 10.3390/microorganisms12061217 (PMC11205819; doi:10.3390/microorganisms12061217)
Supplement: Supplementary file 1 [file microorganisms-12-01217-s001.zip › microorganisms-3058815-supplementary.pdf]

Co-cultivation of Symbiodiniaceae and beneficial bacteria to enhance the growth of coral-symbiotic dinoflagellates.

Yawen Liu[1], Huan Wu[1], Yang Shu[1], Yanying Hua[1], Pengcheng Fu[*] and Jing Liu[*]

[1]State Key Laboratory of Marine Resource Utilization in South China Sea, Hainan university, Haikou 570228, China
[2]International School of Public Health and One Health, Hainan Medical University, Haikou 571199, Hainan, PR China

Correspondence: pcfu@hainanu.edu.cn; liujing66@hainmc.edu.cn

Supplementary materials

Table S1 Target gene and PCR cycle information

Functional trait	Target gene	Forward primer sequence	Reverse primer sequence	Thermocycling
Nitrogen fixation	*nifH*	polF (5′-TGC GAT CCG AAA GCC GAC TC-3′)	polR (5′-ATG GCC ATC ATT TCA CCG GA-3′)	94℃ for 3 min; 30 cycles of 94℃ for 1 min, 55℃ for 1min, and 72℃ for 2min; and final extension cycle of 10min at 72℃
Nitrification	*nirK*	F1aCu (5′-ATC ATG GTC CTG CCG CG-3′)	R3Cu (5′-GCC TCG ATC AGA TTG TGG TT-3′)	94℃ for 2 min; 28 cycles of 94℃ for 30s, 57℃ for 1min, and 72℃ for 1min; and final extension cycle of 10min at 72℃
Denitrification	*nirS*	Cd3aF (5′-GTC AAC GTC AAG GAA ACC GG-3′)	R4cd (5′-CGT TGA ACT TGC CGG TCG G-3′)	94℃ for 2 min; 35 cycles of 94℃ for 30s, 51℃ for 1min, and 72℃ for 1min; and final extension cycle of 10min at 72℃

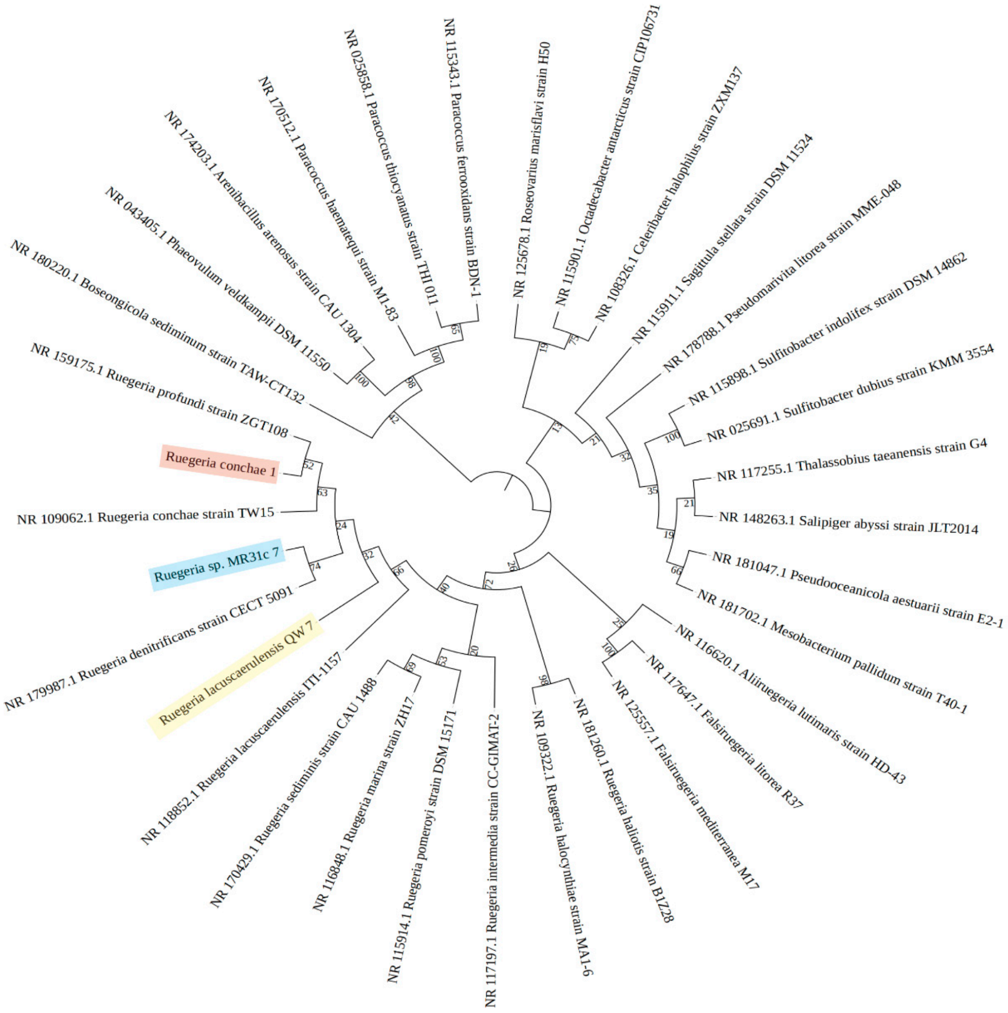

Figure S1 Phylogeny of three stains of *Ruegeria* sp.
